# Frailty and longitudinal pulmonary function trajectories in lung transplant recipients

**DOI:** 10.1186/s12931-026-03924-8

**Published:** 2026-09-25

**Authors:** Chiara Ceolin, Agnese Alessi, Anna Citron, Monica Loy, Mario Virgilio Papa, Anna Bertocco, Tatiana Moro, Stefania Sella, Giuseppe Sergi, Federico Rea, Marina De Rui

**Affiliations:** 1 Department of Neurobiology, Care Sciences and Society, Aging Research Center, Karolinska Institutet and Stockholm University, Stockholm, Sweden; 2 Department of Medicine (DIMED), University of Padua, Padua, Italy; 3 Geriatric Division, Azienda Ospedale-Università Padova, Padua, Italy; 4 Department of Cardiac, Thoracic and Vascular Sciences and Public Health, Thoracic Surgery Unit, University of Padua, Padua, Italy; 5 Department of Biomedical Sciences, Laboratory of Nutrition and Exercise Science, University of Padua, Padua, Italy; 6 Department of Medicine, Clinica Medica 1, Azienda Ospedale-Università Padova, Padua, Italy

**Keywords:** Frailty, Lung transplantation, Pulmonary function, Fried phenotype

## Abstract

**Background:**

Frailty is increasingly recognized as a marker of biological vulnerability associated with adverse clinical outcomes. Lung transplant recipients may be particularly susceptible to frailty, yet its impact on longitudinal pulmonary function improvement after transplantation remains poorly understood.

**Methods:**

Prospective longitudinal study (2-years follow-up) in lung transplant recipients. Physical frailty was assessed using the Fried phenotype. Pulmonary function parameters (vital capacity-VC, forced vital capacity-FVC, forced expiratory volume in 1 s-FEV1, and total lung capacity-TLC) were expressed as percentage of predicted values. Linear mixed-effects models were used to evaluate the association between baseline frailty burden and pulmonary function trajectories over time, adjusting for age, sex, body mass index, underlying respiratory disease category, and time from transplantation to baseline assessment.

**Results:**

A total of 155 lung transplant recipients were included (mean age 48.7 ± 13.3 years; 43.2% female). According to the Fried phenotype, 43.7% were robust, 40.4% pre-frail, and 15.9% frail. Higher frailty burden was independently associated with lower baseline FVC, with borderline associations for VC and FEV1. Significant frailty-by-time interactions were observed for VC (β = 2.03, *p* = 0.010) and FVC (β = 2.20, *p* = 0.003), indicating steeper longitudinal improvement in spirometric values among participants with higher baseline frailty burden. A similar trend was observed for FEV1. Reduced handgrip strength and unintentional weight loss were the frailty components most consistently associated with pulmonary function trajectories.

**Conclusions:**

These findings support the potential role of frailty assessment in identifying vulnerable transplant recipients who may benefit from personalized rehabilitative and multidimensional post-transplant interventions.

**Supplementary Information:**

The online version contains supplementary material available at https://doi.org/10.1186/s12931-026-03924-8.

## Introduction

Frailty is increasingly recognized as a state of reduced physiological reserve and impaired resilience associated with adverse clinical outcomes across a wide range of chronic diseases [1–6]. Although originally conceptualized within geriatrics, frailty is now acknowledged as a relevant determinant of vulnerability beyond older populations, reflecting biological processes such as chronic inflammation, sarcopenia, and multisystem dysregulation [2]. Among available instruments, the Fried physical frailty phenotype is one of the most widely used and validated tools for assessing biological vulnerability [2, 7].

Lung transplant recipients are particularly susceptible to frailty due to the combined effects of advanced respiratory disease, chronic immunosuppressive therapy, infectious complications, and the substantial inflammatory and metabolic burden associated with transplantation [8, 9]. Previous studies have shown that frailty is common in this population and is associated with poorer clinical outcomes and increased postoperative vulnerability [10–14]. However, whether frailty influences longitudinal pulmonary function improvement after transplantation remains unclear.

Therefore, the present study investigated the association between physical frailty and longitudinal pulmonary function trajectories in a cohort of lung transplant recipients. We hypothesized that frailty would be associated with distinct patterns of respiratory change over time and that its assessment could help identify patients who may benefit from targeted post-transplant interventions.

## Materials and methods

### Study design and participants

This prospective longitudinal study included clinically stable lung transplant recipients routinely followed at the University Hospital of Padua. Participant recruitment and eligibility criteria have been previously described [15]. Briefly, adults aged > 20 years who had undergone lung transplantation at least three months earlier were eligible.

Participants underwent baseline assessment (≥ 3 months after transplantation) and, when available, a repeat evaluation approximately two years later.

The study was conducted in accordance with ISHLT ethical principles and the Declaration of Helsinki. The protocol received approval from the local ethics committee (Comitato Etico di Padova; approval number 0014675), and all participants provided written informed consent.

### Study variables

Each participant underwent a clinical and functional assessment at both baseline and follow-up, conducted by qualified medical personnel. This assessment included:

- A comprehensive medical history was systematically collected. Cumulative corticosteroid exposure was calculated considering the entire clinical history, regardless of transplant timing, while time from transplantation was defined as the interval between lung transplantation and study assessment. Comorbidities and disease severity were evaluated using the Cumulative Illness Rating Scale (CIRS) [16]. Functional status was evaluated based on independence in Activities Of Daily Living (ADL) [17].
- Body weight and height were measured using calibrated equipment, and body mass index (BMI) was calculated as kg/m².
- Spirometry was performed by the Respiratory Physiopathology Unit using a calibrated spirometer (Jaeger, Germany) according to manufacturer recommendations. Assessments were conducted in the morning, and the best result from three technically acceptable maneuvers was retained for analysis. The evaluated respiratory parameters included vital capacity (VC), forced vital capacity (FVC), forced expiratory volume in 1 s (FEV1), and total lung capacity (TLC). All measurements were standardized according to international reference equations accounting for age, sex, height, weight, and ethnicity, and were expressed as percentage of predicted values (% predicted).

### Assessment of frailty

Frailty was assessed using the Fried physical frailty phenotype [7], based on the presence of five components: unintentional weight loss, self-reported exhaustion, low physical activity, slow gait speed, and reduced handgrip strength. Participants meeting none of the criteria were classified as robust, those meeting one or two criteria as pre-frail, and those meeting three or more criteria as frail, according to the original Fried criteria.

Unintentional weight loss was defined as self-reported involuntary loss of ≥ 4.5 kg or ≥ 5% of body weight over the previous year. Exhaustion was evaluated through standardized self-report questions derived from the Center for Epidemiologic Studies Depression Scale (CES-D). Low physical activity was assessed using the short form of the Minnesota Leisure Time Physical Activity Questionnaire (MLTPAQ) [18]. Gait speed was measured during a standardized walking test, while muscle strength was evaluated through handgrip dynamometry using the highest value obtained from repeated attempts according to standardized procedures.

### Statistical analyses

Continuous variables were expressed as mean ± standard deviation (SD), while categorical variables were reported as counts and percentages. Differences across frailty categories were assessed using one-way analysis of variance (ANOVA) for continuous variables and chi-square tests for categorical variables. The cross-sectional relationship between frailty burden and pulmonary function at baseline was graphically explored using locally weighted scatterplot smoothing (LOESS) curves. To evaluate the longitudinal association between frailty and pulmonary function trajectories, linear mixed-effects models were fitted for each respiratory parameter (VC, FVC, FEV1, and TLC). Models included random intercepts for participants to account for repeated measurements over time. Fixed effects included age, sex, BMI, underlying respiratory disease, time from transplantation to baseline assessment, years from baseline evaluation, baseline frailty score, and the interaction between frailty score and time. The frailty-by-time interaction term was included to assess whether pulmonary function trajectories differed according to baseline frailty burden. Accordingly, longitudinal trajectories were modeled from the study baseline assessment rather than from the time of transplantation. Model assumptions were assessed by inspection of residual distributions and residual-versus-fitted plots, with no evidence of major violations. Additional exploratory mixed-effects models were performed separately for each individual Fried frailty component (weight loss, exhaustion, low physical activity, slow gait speed, and low handgrip strength) to identify the domains most strongly associated with pulmonary function trajectories. These models were adjusted for the same covariates included in the primary analyses. Regression coefficients (β), standard errors (SE), 95% confidence intervals (CI), and p-values were reported. Mixed-effects models included all available repeated measurements. Statistical significance was defined as a two-sided p-value < 0.05. Statistical analyses were performed using IBM SPSS Statistics software (IBM Corp., Armonk, NY, USA; version 29.0), while graphical visualizations were generated using R software (R Foundation for Statistical Computing, Vienna, Austria; version 4.5.0).

## Results

### Baseline characteristics of the study population

A total of 155 lung transplant recipients (43.2% females) were included in the study (Table 1). The cohort had a mean age of 48.7 ± 13.3 years and a mean BMI of 22.90 ± 4.03 kg/m², with overall preserved functional status. The most common indications for transplantation were cystic fibrosis (30.1%), restrictive lung diseases (22.2%), and obstructive lung diseases (15.7%), while miscellaneous and vascular conditions accounted for 26.8% and 5.2% of cases, respectively. Participants showed substantial exposure to glucocorticoid therapy, with approximately 58% receiving ≥ 10 mg/day at the time of assessment. Time from transplantation to baseline assessment showed a markedly right-skewed distribution, with a median of 14 months (IQR 3–50.25; range 2–270 months). Frailty data were available for 151 participants, among whom 43.7% were classified as robust, 40.4% as pre-frail, and 15.9% as frail according to the Fried phenotype. The mean frailty score was 1.13 ± 1.29. Frail participants were older and showed poorer functional status compared with robust individuals. They were receiving higher corticosteroid doses, whereas no significant differences were observed in comorbidity burden or number of medications (Supplementary Table 1).

**Table 1:** Baseline descriptive characteristics of the total sample

Variable	Total (*n* = 155)
Age (Years)	48.7 (13.3)
Sex, females (%)	67 (43.2%)
BMI (kg/m²)	22.90 (4.03)
ADL	5.84 (0.62)
Primary Condition	
Cystic Fibrosis (%)	46 (30.1%)
Restrictive Lung Diseases (%)	34 (22.2%)
Obstructive Lung Diseases (%)	24 (15.7%)
Miscellaneous (%)	41 (26.8%)
Vascular Diseases (%)	8 (5.2%)
CIRS-CI	4.05 (1.67)
Total Medications Taken	15.05 (3.86)
Duration of Corticosteroid Therapy (Months)	30.19 (47.98)
Current glucocorticoid dosage, *n* (%)	
< 5 mg/day	5 (3.3%)
5–10 mg/day	59 (38.6%)
10–30 mg/day	55 (35.9%)
> 30 mg/day	34 (22.2%)
Frailty phenotype	
Robust	66 (43.7%)
Pre-frail	61 (40.4%)
Frail	24 (15.9%)
Baseline frailty score	1.13 (1.29)

Continuous variables are presented as mean (SD), while categorical variables are reported as counts and percentages. Available data for frailty: *n* = 151. Frailty status was defined according to the Fried phenotype as robust (score = 0), pre-frail (score = 1–2), and frail (score ≥ 3). *Abbreviations* *BMI * body mass index, *ADL * Activities of Daily Living, *CIRS-CI * Cumulative Illness Rating Scale–Comorbidity Index

**Fig. 1:**
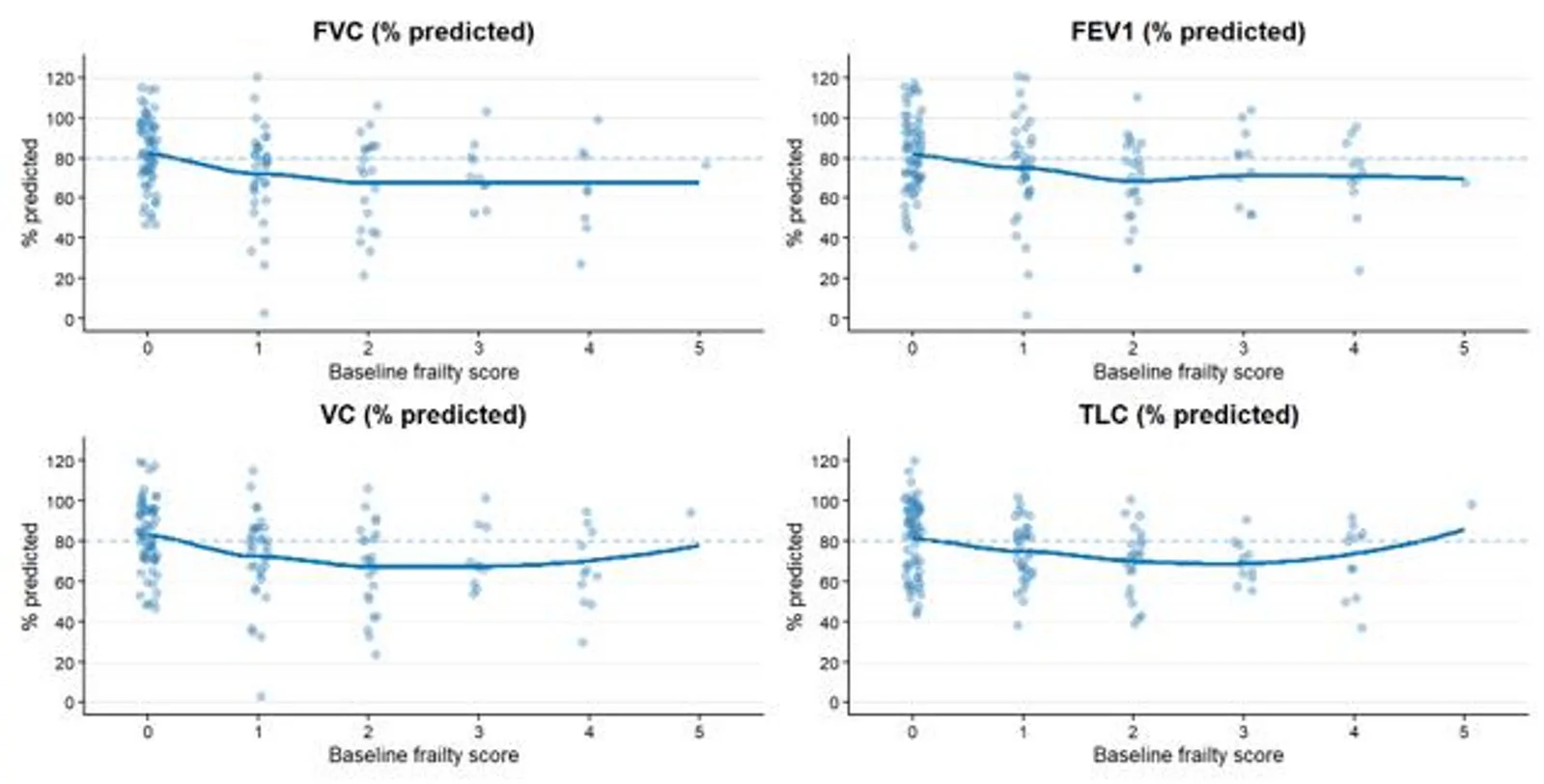
Baseline pulmonary function according to baseline frailty score Notes: Relationship between baseline frailty score and pulmonary function parameters, including forced vital capacity (FVC), forced expiratory volume in 1 second (FEV1), vital capacity (VC), and total lung capacity (TLC), expressed as percentage of predicted values. Each point represents an individual participant at baseline evaluation. Solid lines represent locally weighted smoothing (LOESS) curves used for descriptive visualization of the association between frailty burden and pulmonary function. Dashed horizontal lines indicate the 80% predicted threshold

Baseline pulmonary function was mildly reduced overall, with mean predicted values of 78.5 ± 20.27% for VC, 75.94 ± 20.52% for FVC, 78.0 ± 21.64% for FEV1, and 78.50 ± 17.07% for TLC. Visual inspection of LOESS curves suggested an inverse association between frailty burden and pulmonary function, particularly for dynamic respiratory parameters, without evidence of a clear threshold effect; some flattening was observed at higher frailty scores, where observations were relatively sparse (Fig. 1). Consistently, robust participants showed significantly higher VC, FVC, FEV1, and TLC values compared with pre-frail and frail individuals, whereas differences between pre-frail and frail participants were less evident (Supplementary Table 1).

### Longitudinal association between baseline frailty score and pulmonary function trajectories

Available follow-up pulmonary function assessments were performed after a mean follow-up duration of 17.2 ± 8.9 months. Follow-up pulmonary function data was available for 66 participants. Participants with follow-up data had a more favorable baseline profile, being more frequently robust and less frequently frail (*p* = 0.001), and showing higher baseline VC, FVC, FEV1, and TLC values (all *p* ≤ 0.005) compared with those without follow-up data. Baseline and follow-up pulmonary function parameters are reported in Supplementary Table 2. In the adjusted linear mixed-effects models (Table 2), higher baseline frailty burden was independently associated with lower baseline FVC values, while associations with VC and FEV1 were of borderline statistical significance. Specifically, each additional point in the baseline frailty score was associated with a 3.82%-point lower FVC at baseline. No significant association was observed for TLC. Significant frailty-by-time interactions emerged for VC (β = 2.03, 95% CI 0.50 to 3.55; *p* = 0.010) and FVC (β = 2.20, 95% CI 0.76 to 3.65; *p* = 0.003), indicating steeper longitudinal improvement in spirometric values among participants with higher baseline frailty burden. In practical terms, each additional point in baseline frailty score was associated with approximately 2.0 and 2.2% points greater annual change in VC and FVC, respectively. A similar, although borderline significant, trend was observed for FEV1 (β = 1.73, 95% CI − 0.11 to 3.56; *p* = 0.065), whereas no longitudinal interaction was detected for TLC (β = 0.26, 95% CI − 1.97 to 2.48; *p* = 0.820).

**Table 2:** Linear mixed-effects models evaluating the association between baseline frailty score and longitudinal pulmonary function trajectories

Outcome	Predictor	β (SE)	*p*	95% CI
VC	Follow-up time (years)	1.10 (0.76)	0.153	−0.42 to 2.61
	Baseline frailty score	−2.63 (1.40)	0.061	−5.39 to 0.13
	*Frailty score × years*	*2.03 (0.76)*	***0.010***	*0.50 to 3.55*
FVC	*Follow-up time (years)*	*1.05 (0.77)*	*0.180*	*−0.50 to 2.59*
	*Baseline frailty score*	*−3.82 (1.64)*	***0.021***	*−7.06 to − 0.58*
	*Frailty score × years*	*2.20 (0.72)*	***0.003***	*0.76 to 3.65*
TLC	*Follow-up time (years)*	*0.91 (1.21)*	*0.453*	*−1.50 to 3.32*
	*Baseline frailty score*	*−1.18 (1.17)*	*0.318*	*−3.50 to 1.14*
	*Frailty score × years*	*0.26 (1.12)*	*0.820*	*−1.97 to 2.48*
FEV1	*Follow-up time (years)*	*−0.08 (0.99)*	*0.936*	*−2.05 to 1.89*
	*Baseline frailty score*	*−2.65 (1.55)*	*0.089*	*−5.70 to 0.41*
	*Frailty score × years*	*1.73 (0.92)*	*0.065*	*−0.11 to 3.56*

Models included age, sex, body mass index (BMI), underlying respiratory disease category, time from transplantation to baseline assessment, years from baseline assessment, baseline frailty score, and the interaction between frailty score and time. *Abbreviations* *VC * vital capacity, *FVC * forced vital capacity, *TLC * total lung capacity, *FEV1 * forced expiratory volume in one second

Statistically significant values are reported in bold

### Predicted pulmonary function trajectories according to frailty status

**Fig. 2:**
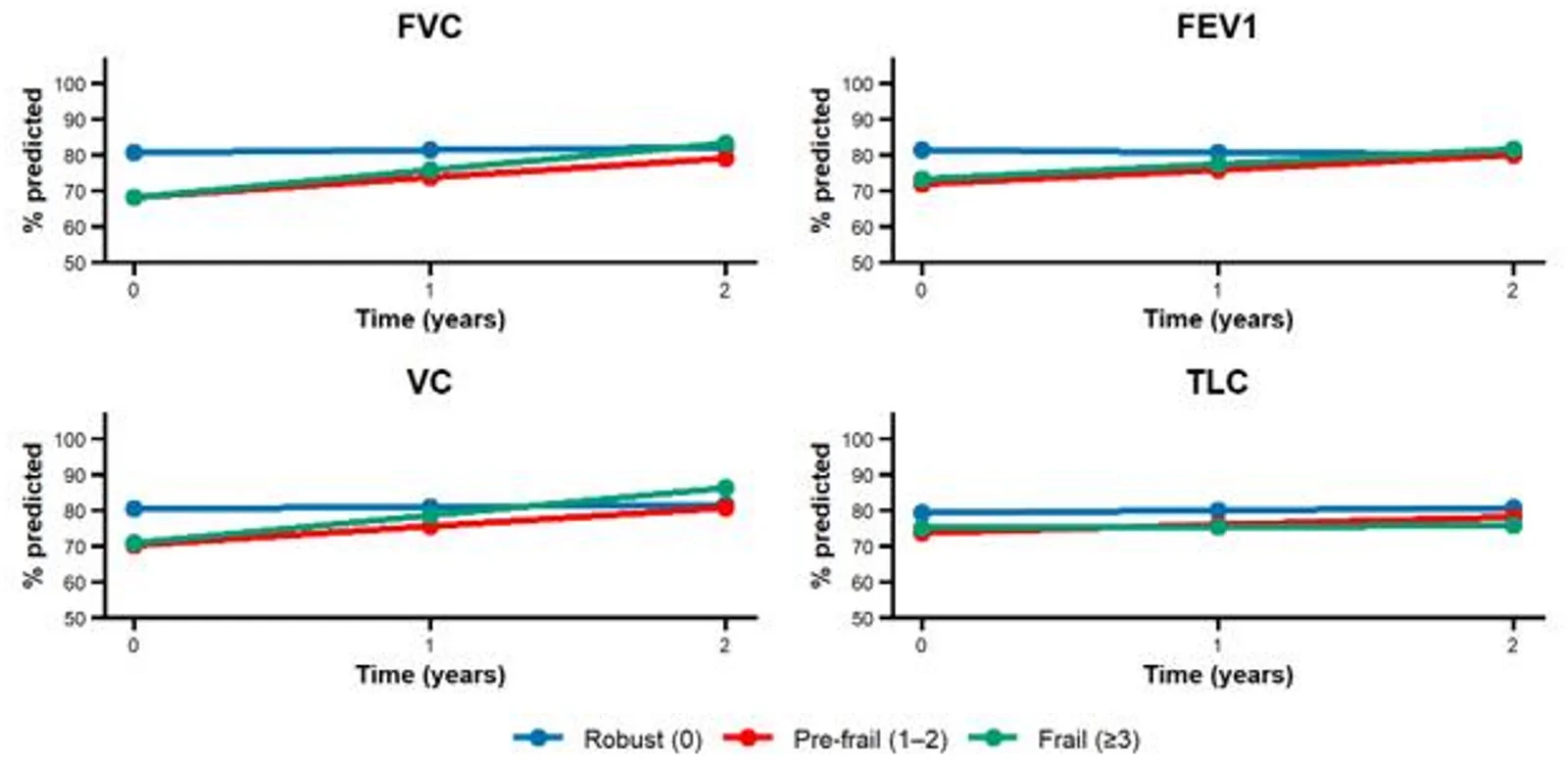
Predicted pulmonary function trajectories according to baseline frailty status Notes: Values were estimated from linear mixed-effects models adjusted for age, sex, BMI, underlying respiratory disease category, and time from transplantation to baseline assessment. Frailty status was categorized according to the Fried phenotype as robust (0 criteria), pre-frail (1-2 criteria), and frail (23 criteria). Predicted trajectories are shown from study baseline to 2 years and assume a linear change over time. FVC: forced vital capacity; FEV1: forced expiratory volume in one second; VC: vital capacity; TLC: total lung capacity

Predicted trajectories according to baseline frailty categories further illustrated these longitudinal patterns (Fig. 2). Pre-frail and frail participants showed lower predicted pulmonary function values at baseline, particularly for VC, FVC, and FEV1, whereas robust individuals maintained consistently higher values over time. However, pre-frail and frail participants exhibited steeper longitudinal improvements during follow-up, resulting in a progressive narrowing of the initial gap between frailty groups, especially for VC and FVC. In contrast, TLC trajectories remained substantially stable and parallel across frailty categories, supporting the absence of a significant frailty-by-time interaction for this parameter.

### Exploratory analyses of individual Fried frailty components

Exploratory item-level analyses suggested that not all Fried components contributed equally to the association between frailty and pulmonary function (Supplementary Table 3). Reduced handgrip strength emerged as the component most consistently associated with impaired pulmonary function trajectories. Participants with low handgrip strength showed significantly lower baseline FVC (β = −9.84, SE = 4.09, 95% CI − 17.92 to − 1.76; *p* = 0.017) and VC (β = −8.48, SE = 3.74, 95% CI − 15.88 to − 1.09; *p* = 0.025), corresponding to approximately 9.8 and 8.5% points lower predicted FVC and VC, respectively, compared with participants without reduced handgrip strength, while the association with FEV1 was of borderline significance (β = −7.31, SE = 4.17, 95% CI − 15.56 to 0.95; *p* = 0.082). Significant positive interactions with time were observed for both FVC (β = 4.26, SE = 1.87, 95% CI 0.52 to 7.99; *p* = 0.026) and FEV1 (β = 4.89, SE = 2.31, 95% CI 0.28 to 9.50; *p* = 0.038), indicating steeper longitudinal slopes for FVC and FEV1 among individuals with lower muscle strength at baseline, corresponding to approximately 4.3 and 4.9% points greater annual change in FVC and FEV1, respectively. Unintentional weight loss was not significantly associated with baseline pulmonary function values but was associated with steeper longitudinal slopes for FVC and FEV1. Specifically, weight loss showed significant positive interactions with time for FVC (β = 4.17, SE = 1.86, 95% CI 0.46 to 7.88; *p* = 0.028) and FEV1 (β = 4.32, SE = 2.06, 95% CI 0.21 to 8.43; *p* = 0.040), corresponding to approximately 4.2 and 4.3% points greater annual change, respectively. Other Fried components, including exhaustion, low physical activity, and slow walking speed, showed weaker and less consistent associations with pulmonary function parameters.

## Discussion

The present study aimed to investigate the longitudinal association between frailty and pulmonary function trajectories in lung transplant recipients. Our findings revealed an apparently counterintuitive pattern: frailer participants, who exhibited significantly lower pulmonary volumes at baseline, showed greater change over time, particularly for FVC, FEV1, and VC. This pattern should nevertheless be interpreted cautiously. Because participants with higher frailty burden had lower pulmonary function values at study baseline, regression to the mean and greater room for improvement may have contributed to the observed frailty-by-time interactions. Conversely, participants with better baseline pulmonary function may have had less potential for further improvement because of proximity to physiological ceilings. Exploratory analyses of individual Fried phenotype components further suggested that reduced muscle strength and unintentional weight loss were the frailty domains most strongly associated with pulmonary function trajectories. Overall, our findings suggest that baseline frailty burden is associated with distinct longitudinal pulmonary function trajectories in lung transplant recipients.

Our frail participants were predominantly affected by obstructive and restrictive respiratory diseases, in line with previous literature reporting a high burden of frailty in chronic respiratory conditions [19, 20]. Although a reduction in pulmonary volumes could partly be expected as a consequence of the underlying respiratory disease itself, in our cohort all pulmonary function parameters were already reduced at baseline not only in frail but also in pre-frail individuals. This finding suggests that the observed impairment may not be exclusively attributable to the primary lung disease, but could also reflect the systemic biological vulnerability associated with frailty. This hypothesis is biologically plausible. COPD, for instance, shares several pathophysiological mechanisms with frailty, including aging, smoking exposure, chronic inflammation, endocrine dysregulation, and skeletal muscle impairment [21]. On the other hand, restrictive respiratory diseases are frequently characterized by early activity-limiting dyspnea, exercise intolerance, and debilitating fatigue, all factors potentially contributing to reduced physical reserve and frailty development [21]. In addition, pre-frail and frail participants in our cohort were receiving higher corticosteroid doses, despite no significant differences in the timing of post-transplant assessment across frailty categories. Therefore, the observed differences cannot simply be explained by a longer post-transplant disease course. This aspect is particularly relevant considering that patients with cystic fibrosis — who represented the majority of robust participants in our cohort — are often transplanted earlier in life compared with patients affected by obstructive or restrictive chronic respiratory diseases, which typically manifest later. Consequently, corticosteroid exposure itself may have contributed to the observed association between frailty and impaired pulmonary function. Indeed, long-term or high-dose corticosteroid therapy has been shown to accelerate frailty development through multiple mechanisms, including skeletal muscle wasting, reduced bone mineral density, and increased susceptibility to infections, particularly in individuals with chronic inflammatory conditions [22].

Our study adds an important and novel perspective to the existing literature on frailty and respiratory function. To date, most available evidence has primarily focused on the cross-sectional association between pulmonary function impairment and frailty, showing, for example, that lower FEV1 and FVC values are associated with a higher prevalence of frailty in community-dwelling older adults [23]. Other studies have investigated longitudinal pulmonary decline in relation to frailty in chronic respiratory diseases, demonstrating significantly different trajectories of FEV1/FVC and FEF25–75% among older patients with COPD according to frailty status [24]. Additional evidence has mainly addressed the prevalence and clinical burden of frailty in COPD populations [25] or the association between respiratory performance indicators such as PEF % predicted and frailty risk in middle-aged and older adults [26]. Within the lung transplant setting, previous studies have primarily examined frailty before transplantation as a predictor of subsequent outcomes, or post-transplant frailty in relation to outcomes such as mortality, quality of life, and allograft dysfunction [10–14]. In contrast, our study shifts both the clinical setting and the conceptual perspective. To the best of our knowledge, this is the first study specifically investigating longitudinal pulmonary function trajectories in relation to frailty among lung transplant recipients, a population characterized by profound biological vulnerability but also by substantial potential for functional recovery. Rather than focusing exclusively on frailty prevalence or static pulmonary impairment, our findings suggest that frailty is associated with the dynamic process of respiratory change after transplantation, particularly with respect to dynamic pulmonary parameters over time. Importantly, these associations remained evident even after adjustment for the underlying respiratory disease category, suggesting that the observed relationship may not be exclusively explained by the primary pulmonary condition itself. The findings are biologically plausible, as dynamic respiratory volumes are inherently dependent on respiratory muscle performance and expiratory effort. Several mechanisms may underlie this association, including sarcopenia [27], chronic inflammation [28], physical deconditioning, and impaired physiological reserve [29], all of which are closely linked to frailty and may negatively affect ventilatory efficiency and respiratory muscle function. The absence of a significant association between frailty and longitudinal changes in TLC, in contrast to VC, FVC, and, to a lesser extent, FEV1, may further support a predominant involvement of the dynamic components of respiratory function. Dynamic pulmonary measures are particularly influenced by respiratory muscle performance, expiratory effort, and the ability to generate and sustain an effective respiratory maneuver [30], and may therefore be more sensitive to the muscular deconditioning and reduced physiological reserve associated with frailty. Conversely, TLC reflects overall lung volume [31] and may be less sensitive to changes in respiratory muscle performance. Moreover, because TLC represents the sum of different lung volume compartments, changes in individual components may not necessarily translate into detectable changes in total lung capacity [32]. Notably, reduced handgrip strength was among the Fried phenotype components most consistently associated with pulmonary function trajectories in our cohort, particularly for FVC and FEV1. Handgrip strength is a well-established marker of global muscle strength [33], and it is therefore possible that the lower handgrip values observed in frail participants may also reflect impaired inspiratory and expiratory muscle performance. Similarly, unintentional weight loss — another frailty component associated with longitudinal pulmonary changes — may represent the clinical expression of systemic catabolic processes, muscle wasting, and reduced metabolic reserve. Chronic respiratory diseases and frailty also share several pathophysiological pathways, including inflammation, oxidative stress, endocrine dysregulation, and reduced physical activity [34–36], potentially creating a vicious cycle of dyspnea, inactivity, muscle decline, and worsening functional reserve. Following transplantation, however, the combination of improved respiratory mechanics provided by the transplanted lung and the rehabilitative pathways routinely implemented after surgery may contribute to a progressive recovery of respiratory performance. Importantly, the clinical significance of the observed longitudinal changes in pulmonary function should be interpreted cautiously. Although frailer participants showed significantly steeper improvements in VC and FVC over time, the present study was not designed to determine whether these changes translated into clinically meaningful benefits in terms of exercise capacity, respiratory symptoms, hospitalization rates, or quality of life. Therefore, the observed trajectories should primarily be interpreted as evidence of differential pulmonary function change according to baseline frailty burden rather than as direct evidence of improved clinical outcomes. Future studies integrating pulmonary function with patient-centered and functional outcomes are needed to establish the clinical relevance of these findings.

From a clinical perspective, these findings highlight two important aspects. First, pre-frail participants appeared to show pulmonary function trajectories broadly similar to those observed in frail individuals. This suggests that systematic frailty screening in lung transplant recipients may help identify a subgroup of patients — namely pre-frail individuals — who are often overlooked in clinical practice despite potentially representing a vulnerable population that could benefit from closer monitoring and targeted interventions aimed at optimizing post-transplant outcomes. Second, our findings suggest that frailty should not necessarily be interpreted as a marker of irreversible functional decline, as recipients with greater frailty burden still demonstrated substantial improvement in pulmonary function over time. This interpretation is also consistent with the dynamic nature of frailty, which may evolve over time rather than representing a fixed patient characteristic. This raises the possibility that frailty assessment should not be considered exclusively as a prognostic tool, but also as a means of identifying potentially modifiable vulnerabilities that may respond to personalized multidisciplinary strategies, including rehabilitation, nutritional support, and physical interventions. In this perspective, integrating frailty assessment into post-transplant care pathways may help optimize long-term functional recovery and patient-centered outcomes. Although our data does not identify a specific optimal time point for frailty assessment after transplantation, the observed associations support its potential value during longitudinal post-transplant follow-up, particularly for identifying pre-frail and frail recipients who may benefit from closer monitoring.

### Limitations and strengths

Several limitations should be acknowledged. First, this was a single-center study, potentially limiting the generalizability of the findings. In addition, the number of participants classified as frail was relatively small, which may have reduced the statistical power of some analyses. Furthermore, follow-up data were not available for all participants, and those with follow-up measurements had lower frailty burden and better baseline pulmonary function, potentially introducing attrition-related selection into the longitudinal estimates. Pre-transplant pulmonary function and frailty data were also unavailable, limiting the possibility of evaluating individual functional trajectories before and after transplantation. Although models were adjusted for underlying respiratory disease, residual confounding related to functional status, corticosteroid exposure, and post-transplant clinical course cannot be excluded. In particular, detailed longitudinal information on relevant post-transplant complications and treatments, including acute rejection, chronic lung allograft dysfunction (CLAD), acute infections, pulmonary rehabilitation, and changes in immunosuppressive therapy over time, was not available. Moreover, patient-centered outcomes such as exercise capacity, respiratory symptoms, hospitalization rates, and quality of life were not systematically assessed, preventing us from determining whether the observed pulmonary function improvements translated into clinically meaningful benefits. Finally, the exploratory analyses performed on individual Fried frailty components should be considered hypothesis-generating.

Despite these limitations, the study also has important strengths. Frailty was assessed using the Fried phenotype, one of the most validated and widely adopted instruments for the evaluation of physical frailty. In addition, pulmonary function was assessed through standardized spirometric parameters repeatedly collected over time, allowing a dynamic evaluation of respiratory trajectories. Moreover, the longitudinal design represents a major strength, as studies investigating the relationship between frailty and pulmonary function trajectories in lung transplant recipients remain scarce.

### Conclusions

In conclusion, higher baseline frailty burden was associated with lower pulmonary function at study baseline and with distinct longitudinal trajectories during follow-up, characterized by steeper changes in VC and FVC. Frailty assessment may help identify recipients with different patterns of pulmonary function change who could benefit from personalized multidimensional interventions. Further studies are needed to clarify the underlying biological mechanisms and clinical significance of these trajectories.

## Supplementary Information

Supplementary Material 1.

## Data Availability

The datasets generated and/or analyzed during the current study are available from the corresponding author on reasonable request.
