# Supplementary material for: Frailty and longitudinal pulmonary function trajectories in lung transplant recipients
Source: Respir Res. 2026 Sep 25;27:369. doi: 10.1186/s12931-026-03924-8 (PMC13613669; doi:10.1186/s12931-026-03924-8)
Supplement: Supplementary file 1 [file 12931_2026_3924_MOESM1_ESM.docx]

**Supplementary Table 1. Baseline clinical characteristics and pulmonary function parameters according to frailty status**

| *Variable* | *Robust (n=66)* | *Pre-frail (n=61)* | *Frail (n=24)* | *p-value* |
| --- | --- | --- | --- | --- |
| *Age (years)* | *45.2 ± 11.9* | *50.2 ± 14.0* | *57.1 ± 11.2* | *<0.001* |
| *Sex, females (%)* | *31 (47.0%)* | *40 (65.6%)* | *15 (62.5%)* | *0.089* |
| *BMI (kg/m²)* | *23.8 ± 4.3* | *22.3 ± 3.7* | *22.1 ± 4.2* | *0.066* |
| *ADL* | *6.0 ± 0.0* | *5.9 ± 0.7* | *5.3 ± 1.0* | *<0.001* |
| *Primary condition, n (%)* |  |  |  | *<0.001* |
| *Cystic fibrosis* | *33 (50.8%)* | *11 (18.3%)* | *1 (4.2%)* |  |
| *Obstructive lung diseases* | *6 (9.2%)* | *13 (21.7%)* | *5 (20.8%)* |  |
| *Restrictive lung diseases* | *8 (12.3%)* | *19 (31.7%)* | *7 (29.2%)* |  |
| *Miscellaneous* | *17 (26.2%)* | *13 (21.7%)* | *8 (33.3%)* |  |
| *Vascular diseases* | *1 (1.5%)* | *4 (6.7%)* | *3 (12.5%)* |  |
| *Time from transplantation to baseline assessment (months)* | *57.1 ± 45.9* | *23.8 ± 44.7* | *43.5 ± 175.2* | *0.070* |
| *CIRS-CI* | *4.17 ± 1.59* | *4.02 ± 1.77* | *3.79 ± 1.79* | *0.641* |
| *Total medications taken* | *15.7 ± 4.2* | *14.6 ± 3.7* | *14.3 ± 3.4* | *0.168* |
| *Current glucocorticoid dosage, n (%)* |  |  |  | *<0.001* |
| *<5 mg/day* | *4 (6.2%)* | *0 (0.0%)* | *1 (4.2%)* |  |
| *5–10 mg/day* | *41 (63.1%)* | *14 (23.3%)* | *4 (16.7%)* |  |
| *10–30 mg/day* | *15 (23.1%)* | *26 (43.3%)* | *11 (45.8%)* |  |
| *>30 mg/day* | *5 (7.7%)* | *20 (33.3%)* | *8 (33.3%)* |  |
| *Pulmonary function parameters (% predicted)* |  |  |  |  |
| *VC* | *83.3 ± 17.6* | *70.5 ± 22.1* | *70.0 ± 17.9* | *0.001* |
| *FVC* | *82.4 ± 17.2* | *70.4 ± 23.2* | *69.5 ± 18.7* | *0.002* |
| *FEV1* | *82.4 ± 19.7* | *72.4 ± 24.0* | *73.3 ± 18.7* | *0.027* |
| *TLC* | *81.5 ± 18.3* | *73.2 ± 15.7* | *71.6 ± 15.3* | *0.008* |

*Notes: Continuous variables are presented as mean ± standard deviation (SD), while categorical variables are expressed as counts and percentages within frailty categories. P-values were obtained using one-way ANOVA or chi-square tests, as appropriate. Frailty status was defined according to the Fried phenotype as robust (0 criteria), pre-frail (1–2 criteria), and frail (≥3 criteria). Abbreviations: BMI, body mass index; ADL, Activities of Daily Living; CIRS-CI, Cumulative Illness Rating Scale–Comorbidity Index; VC, vital capacity; FVC, forced vital capacity; FEV1, forced expiratory volume in 1 second; TLC, total lung capacity.*

**Supplementary Table 2. Changes in pulmonary function parameters over follow-up**

| **Parameter** | **Baseline** | **Follow-up** |
| --- | --- | --- |
| **VC (% pred)** | 78.50 (20.27) | 84.34 (22.38) |
| **FVC (% pred)** | 75.94 (20.52) | 86.17 (20.22) |
| **FEV1 (% pred)** | 78.0 (21.64) | 84.61 (22.01) |
| **TLC (% pred)** | 78.50 (17.07) | 82.14 (20.24) |

*Values are presented as mean (standard deviation). Follow-up pulmonary function data were available for 69 participants. Abbreviations: FVC, forced vital capacity; FEV1, forced expiratory volume in 1 second; VC, vital capacity; TLC, total lung capacity.*

**Supplementary Table 3. Exploratory item-level mixed models of Fried frailty components and pulmonary function trajectories**

| *Outcome* | *Fried component* | *Component β (SE)* | *p* | *95% CI* | *Component × years β (SE)* | *p* | *95% CI* |
| --- | --- | --- | --- | --- | --- | --- | --- |
| *FVC* | *Weight loss* | *−5.73 (4.06)* | *0.161* | *−13.76 to 2.30* | *4.17 (1.86)* | ***0.028*** | *0.46 to 7.88* |
|  | *Exhaustion* | *−5.63 (5.21)* | *0.282* | *−15.94 to 4.68* | *4.24 (2.53)* | *0.099* | *−0.82 to 9.29* |
|  | *Low physical activity* | *−9.13 (6.14)* | *0.140* | *−21.28 to 3.02* | *5.82 (2.99)* | *0.056* | *−0.16 to 11.80* |
|  | *Slow walking speed* | *−3.34 (5.46)* | *0.543* | *−14.14 to 7.47* | *—* | *—* | *—* |
|  | *Low handgrip strength* | *−9.84 (4.09)* | ***0.017*** | *−17.92 to −1.76* | *4.26 (1.87)* | ***0.026*** | *0.52 to 7.99* |
| *FEV1* | *Weight loss* | *−4.79 (4.11)* | *0.246* | *−12.92 to 3.34* | *4.32 (2.06)* | ***0.040*** | *0.21 to 8.43* |
|  | *Exhaustion* | *−4.95 (5.19)* | *0.342* | *−15.20 to 5.30* | *0.88 (3.05)* | *0.773* | *−5.19 to 6.96* |
|  | *Low physical activity* | *−6.85 (6.40)* | *0.286* | *−19.49 to 5.79* | *3.68 (3.78)* | *0.333* | *−3.85 to 11.21* |
|  | *Slow walking speed* | *−5.15 (5.38)* | *0.340* | *−15.79 to 5.49* | *1.53 (11.12)* | *0.891* | *−20.66 to 23.71* |
|  | *Low handgrip strength* | *−7.31 (4.17)* | *0.082* | *−15.56 to 0.95* | *4.89 (2.31)* | ***0.038*** | *0.28 to 9.50* |
| *VC* | *Weight loss* | *−3.63 (3.82)* | *0.343* | *−11.18 to 3.91* | *4.54 (2.64)* | *0.089* | *−0.70 to 9.79* |
|  | *Exhaustion* | *−5.62 (4.87)* | *0.250* | *−15.24 to 3.99* | *4.36 (4.09)* | *0.289* | *−3.76 to 12.48* |
|  | *Low physical activity* | *−7.36 (5.83)* | *0.209* | *−18.89 to 4.16* | *5.97 (3.65)* | *0.106* | *−1.29 to 13.22* |
|  | *Slow walking speed* | *−1.75 (4.86)* | *0.719* | *−11.36 to 7.86* | *1.79 (8.73)* | *0.838* | *−15.64 to 19.22* |
|  | *Low handgrip strength* | *−8.48 (3.74)* | ***0.025*** | *−15.88 to −1.09* | *3.11 (1.85)* | *0.097* | *−0.58 to 6.80* |

*Notes. Each Fried component was entered in a separate linear mixed-effects model adjusted for age, sex, BMI, underlying respiratory disease category, time from transplantation to baseline assessment, years from baseline, and the interaction between the component and years. For slow walking speed in the FVC model, the interaction term was not estimated because it was redundant. FVC: forced vital capacity; FEV1: forced expiratory volume in one second; VC: vital capacity.*
